# Hybridization Effect on Interlaminar Bond Strength, Flexural Properties, and Hardness of Carbon–Flax Fiber Thermoplastic Bio-Composites

**DOI:** 10.3390/polym15244619

**Published:** 2023-12-05

**Authors:** Mohsen Bahrami, Juan Carlos del Real, Mahoor Mehdikhani, José Antonio Butenegro, Juana Abenojar, Miguel Ángel Martínez

**Affiliations:** 1 Materials Science and Engineering and Chemical Engineering Department, University Carlos III de Madrid, 28911 Leganes, Spain; jbuteneg@ing.uc3m.es (J.A.B.); abenojar@ing.uc3m.es (J.A.); mamc@ing.uc3m.es (M.Á.M.); 2 Institute for Research in Technology, Mechanical Engineering Department, ICAI, Universidad Pontificia Comillas, 28015 Madrid, Spain; delreal@comillas.edu; 3 Department of Materials Engineering, KU Leuven, 3001 Leuven, Belgium; mahoor.mehdikhani@kuleuven.be

**Keywords:** hybrid thermoplastic bio-composite, flax fibers, PA11, interlaminar bond strength, hardness, flexural properties

## Abstract

Hybridizing carbon-fiber-reinforced polymers with natural fibers could be a solution to prevent delamination and improve the out-of-plane properties of laminated composites. Delamination is one of the initial damage modes in composite laminates, attributed to relatively poor interlaminar mechanical properties, e.g., low interlaminar strength and fracture toughness. This study examined the interlaminar bond strength, flexural properties, and hardness of carbon/flax/polyamide hybrid bio-composites using peel adhesion, three-point bending, and macro-hardness tests, respectively. In this regard, interlayer hybrid laminates were produced with a sandwich fiber hybrid mode, using woven carbon fiber plies (C) as the outer layers and woven flax fiber plies (F) as the inner ones (CFFC) in combination with a bio-based thermoplastic polyamide 11 matrix. In addition, non-hybrid carbon and flax fiber composites with the same matrix were produced as reference laminates to investigate the hybridization effects. The results revealed the advantages of hybridization in terms of flexural properties, including a 212% higher modulus and a 265% higher strength compared to pure flax composites and a 34% higher failure strain compared to pure carbon composites. Additionally, the hybrid composites exhibited a positive hybridization effect in terms of peeling strength, demonstrating a 27% improvement compared to the pure carbon composites. These results provide valuable insights into the mechanical performance of woven carbon–flax hybrid bio-composites, suggesting potential applications in the automotive and construction industries.

## 1. Introduction

Hybrid composites made of carbon and natural fibers, such as flax, jute, hemp, and bamboo, have gained increasing attention in recent years due to their unique combination of properties [1,2]. These composites have the potential to offer high strength and stiffness, as well as improved environmental sustainability compared to traditional composite materials [3]. Among natural fibers, flax fibers are recognized for their significant potential as a reinforcement material in composites, attributed to their high tensile strength, lightweight nature, and biodegradability [4,5,6]. Moreover, flax fibers have good chemical compatibility with carbon fibers and resin systems, making them an ideal candidate for hybridization with carbon fibers. The market for bio-composites, which includes robust bast fibers like hemp and flax, is anticipated to experience a substantial increase with a compound annual growth rate (CAGR) of 14.20%, from USD 21 billion in 2020 to USD 61 billion in 2028 [7].

In recent years, high-performance glass and carbon fibers have been the most applicable synthetic fibers for hybridizing natural fiber composites. Carbon fibers, featuring a diverse range of aspect ratios along with low thermal expansion, a light weight, high specific strength and stiffness, chemical resistance, and temperature tolerance, are utilized as multifunctional reinforcements in carbon-fiber-reinforced polymer composites (CFRPs) [8,9]. Glass fibers, known for their affordability, high tensile strength, chemical resistance, and excellent insulating properties, are employed extensively in the production of the most prevalent category of composite materials known as glass-fiber-reinforced polymer composites (GFRPs) [10,11]. Table 1 compares the physical and mechanical properties of some natural and synthetic fibers.

The market for CFRPs is projected to experience a CAGR of 8.8% from 2022 to 2032, in comparison to GFRPs, which are anticipated to have a CAGR of 6% [12,13]. The increased utilization of CFRP composites in aerospace and defense, along with the demand for corrosion-resistant materials for concrete reinforcement, serve as key factors contributing to the market’s expansion. This estimation suggests that the CFRP market is expected to represent approximately 3–5% of the total global reinforced plastics market [12].

**Table 1:** Mechanical properties of some natural and synthetic fibers.

Fiber	Density (g/cm^3^)	Tensile Strength (MPa)	E-Modulus (GPa)	Elongation at Break (%)	Ref.
**Bast fiber**					
Flax	1.5	345–1100	27.6	0.2–3.2	[14,15,16,17]
Hemp	1.48	690	30–70	1.6–4	[14,15,16,18]
Jute	1.3–1.45	393–773	10.0–30.0	1.2–1.8	[14,15,16,17]
Kenaf	-	930	22.0–60.0	1.6	[14,16,17]
**Leaf fiber**					
Sisal	1.5	468–640	9.4–22.0	3.0–7.0	[14,15,19,20]
Curaua	1.4	500–1150	9–11.8	3.7–7.5	[14,16,17]
Pineapple	1.5	413–1627	34.5–82.5	0.8–1.6	[17,21,22,23]
**Fruit/Seed fiber**					
Cotton	1.5–1.6	287–800	5.5–12.6	7.0–8.0	[16,17,19]
Coir	1.2	131–175	4.0–6.0	15.0–40	[14,16,17,19]
Oil palm	0.7–1.55	248	3.2	25.0	[14,16,17,24]
**Synthetic fiber**					
Aramid	1.4	3000–3150	63.0–67.0	3.3–3.7	[15,17,19]
Carbon	1.7	4000	230–240	1.4–1.8	[15,17,19]
E-glass	2.5	2000–3500	70.0	2.5	[15,19,21,25]
S-glass	2.5	4570	86.0	2.8	[15,17,19,21]

Among these natural and synthetic fibers, flax, in hybrid form with carbon fibers, has recently been found to offer a distinct set of characteristics, regardless of the matrix being thermoplastic or thermoset. These hybrid composites offer a high strength-to-weight ratio, excellent stiffness and impact resistance, and low environmental impact compared to traditional carbon composites [26,27,28]. The carbon fibers provide high stiffness and strength, while the flax fibers offer superior impact resistance [29,30], damping properties [31,32], and sustainability [33]. Combining these fibers results in a hybrid composite that can be tailored to meet specific requirements. The development of these composites is expected to have significant applications in various industries, including the construction, automotive, and sporting goods industries.

The applications of hybrid composites made of flax and carbon fiber are diverse. Some of the potential applications include:

- Automotive: carbon–flax hybrid composites are applicable in the automotive industry to reduce the weight of vehicles and hence their fuel consumption [30,31,34].
- Sporting goods: carbon–flax hybrid composites can be used to manufacture high-performance sporting goods such as bicycles, snowboards, and tennis rackets, for which the strength, damping, and weight are critical [35,36,37].
- Marine: carbon–flax hybrid composites have applicability in the marine industry to manufacture boat hulls, decks, and other components demanding exceptional strength and durability [38,39].
- Construction: carbon–flax hybrid composites find utility in the construction industry for manufacturing structural elements like beams and columns, known for their light weight, robust mechanical characteristics, and resistance to environmental wear and degradation [40,41].
- Renewable energy: carbon–flax hybrid composites have practical applications in wind turbine blades and solar panels due to their lightweight nature and durability [42,43].

Several studies have investigated the mechanical properties of hybrid composites made of flax and carbon fibers. Wang et al. [44] achieved a positive hybrid effect in terms of tensile properties by manufacturing a hybrid epoxy composite consisting of flax skins and a carbon core produced with a wet winding technique. Karacor et al. [45] improved the tensile strength and hardness of pure flax/epoxy composites by incorporating carbon fabric layers between the flax layers, with an alternating sequence. In another research endeavor, Kumar et al. [46] studied the mechanical behavior of flax/carbon/polyvinyl butyral hybrid composites with a varying fiber content and layer sequencing. They proved that the fatigue lifetime increased for the carbon/flax hybrid composites with an increment in the content of carbon in comparison to the content of flax fibers. 

Many researchers [27,28,41,47] have confirmed the importance of the outer layers in influencing the tensile properties, while the middle layers have a minimal effect. Nisini et al. [48] observed a similar positive effect of hybridization on flexural properties and interlaminar shear strength when unidirectional flax fabric layers were used as the inner plies and woven carbon fibers as the outer plies. It was found that this layup configuration reduced the tendency of flax fibers to bend, enhancing their rigidity, which was beneficial for both the flexural and interlaminar strength of the hybrid composites. Sarasini et al. [49] compared hybrid composites of flax and carbon fibers with two different stacking sequences. They reported that placing the flax layers on the outside improved impact absorption and hindered crack propagation in the laminate. 

One purpose of hybridizing carbon-fiber-reinforced polymer composites is to improve their interlaminar bond strength, thereby reducing delamination. Delamination, which is a type of layer decohesion in laminated composite materials, is one of the most common damage modes and is still a challenging problem in hybrid composites [50]. Delamination in carbon-fiber-reinforced polymer laminates is primarily governed by the interlaminar bond strength, which reflects the adhesive properties between adjacent composite layers. Researchers commonly evaluate this property through various interlaminar fracture tests, such as the double cantilever beam [51,52], end-notched flexure [53,54], and peeling tests [55,56]. These tests provide valuable insights into the propensity for delamination within composite structures.

In hybrid composites of flax and carbon fibers, there are differences in the properties of the two fibers. These differences exist, for example, in their coefficient of thermal expansion, moisture absorption, and surface chemistry, which can result in issues like poor bonding between the layers and (hence) delamination. 

A low interlaminar bond strength can lead to a reduced load-carrying capacity, decreased fatigue life, and increased susceptibility to damage in composite structures [57]. This issue can be especially problematic in high-performance applications where the composite structure is subjected to severe loading conditions. Various approaches have been developed to improve the interlaminar bond strength of laminated carbon/natural fiber hybrid composites. For example, researchers have explored the use of interlayers [58,59,60], fiber surface treatments [61,62,63], and coupling agents [64,65,66] to improve the adhesion between the two fiber types.

The interlaminar bond strength of laminated composites can significantly impact the flexural and hardness properties of the material. When a load is applied to a composite laminate, it is distributed between the layers. If the interlaminar bond strength is weak, the layers may separate, leading to the local loss of load carrying and hence stress concentrations, decreasing the hardness and flexural strength.

**Figure 1:**
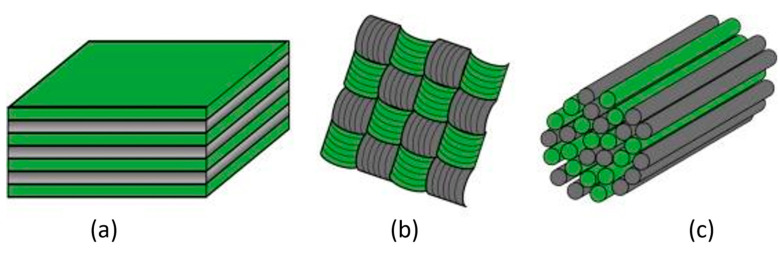
The three main hybrid configurations: (**a**) interlayer, (**b**) intralayer, and (**c**) intrayarn (colors represent two different fiber types) [67].

Regarding the hybridization mode, fibers in hybrid composites can be combined in various configurations, with the three most significant ones depicted in Figure 1 [67]. In the interlayer configuration (Figure 1a), layers of two fiber types are stacked on top of each other. In the intralayer hybrid configuration (Figure 1b), the two fiber types are blended within the layers, as shown by the different yarns co-woven into a fabric. Alternatively, the two fiber types can be mixed or comingled at the fiber level, resulting in an intrayarn hybrid (Figure 1c). In this study, the interlayer configuration was chosen primarily in response to constraints imposed by the characteristics of the selected fiber types and the complexities of the manufacturing method. This decision was strategically aligned with considerations of simplicity and cost-effectiveness, principles crucial for optimizing the inherent efficiency of the manufacturing process. By systematically stacking layers of distinct fiber types—flax, carbon, and polyamide 11 (PA11)—this approach ensured a controlled and methodical distribution within the composite structure.

This study examined how hybridizing carbon plies with flax fabrics in an interlayer sandwich hybrid mode affected the interlaminar bond strength, flexural properties, and hardness during peeling, three-point bending, and macro-hardness tests. Previous research in this area has predominantly used epoxy resin, while in the current study, a bio-based PA11 matrix was used. The choice of polyamide resin, particularly the bio-based PA11 matrix, was made based on the careful consideration of its unique properties and environmental advantages. Aliphatic polyamides, such as PA11, represent a paramount class of engineering thermoplastic polymers renowned for their outstanding material characteristics within the industry [68]. These materials have been extensively researched across diverse industrial sectors, including the automotive, textile, packaging, electric and electronics, sports, and oil and gas industries, owing to their exceptional combination of properties, including excellent durability [69] and mechanical strength [70], high-temperature and chemical resistance [71], ease of processing, and high melting point [72]. Notably, recent years have witnessed a significant surge in the demand for PA products, especially for replacing specific metal structures in power tools, automobiles, and powertrain systems [73]. In particular, PA11 demonstrates superior toughness when compared to other bio-based thermoplastic resins such as poly(lactic acid), a commonly suggested matrix for bio-composites [74,75]. There is a notable gap in the existing literature regarding understanding the mechanical characteristics of carbon–flax hybrid thermoplastic bio-composites. This study addressed this gap by conducting comprehensive mechanical analyses of a specific carbon–flax hybrid bio-composite system.

In addition to the abovementioned applications for carbon–flax hybrid composites, typically employing epoxy resin, our hybrid bio-composite with a PA11 matrix introduces new possibilities in various domains. The unique properties of our material open avenues for applications in the semi-structural components found inside aircraft, automobiles, and habitats [76,77]. Furthermore, the cosmetic, stamping, transport, luxury, sport and leisure, and home sectors [77,78] stand out as potential areas where our hybrid bio-composite could offer distinct advantages over their traditional counterparts.

## 2. Materials and Methods

### 2.1. Materials

The carbon and flax fiber woven fabrics were produced by Castro Composites S.L. (Pontevedra, Spain) and Lucio J&M (Madrid, Spain), respectively, with a 2 × 2 twill pattern and an areal density of 200 g/m^2^. Fibers were stored at room temperature and relative humidity (approximately 23 °C and 33%, respectively) before the fabrication of the composite materials. Table 2 shows the fiber properties according to the supplier datasheets. Lower mechanical properties were noted for flax, as it is a natural fiber. The thermoplastic matrix was a commercial bio-based PA11, which was obtained from Arkema (Madrid, Spain). PA sheets were prepared from pellets with a hot-press, following the procedure established in our previous work [79].

### 2.2. Surface Treatment

**Figure 2:**
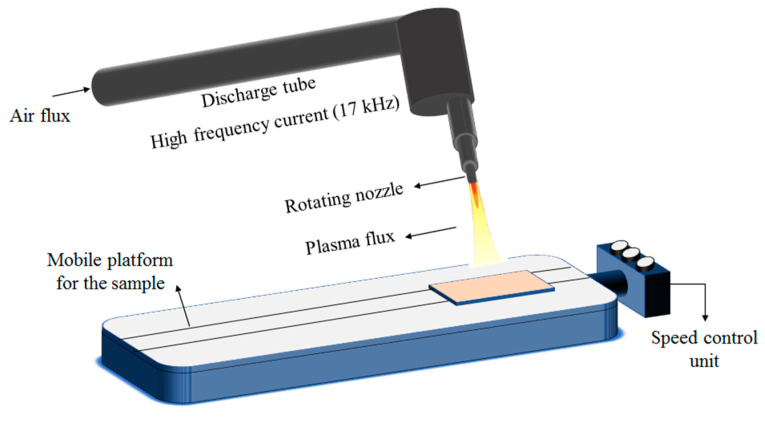
Schematic of the atmospheric-pressure plasma torch device [81].

To enhance the adhesion between the flax fabrics and the PA11 matrix, a surface treatment using an atmospheric pressure plasma torch from Plasma Treat GmbH (Steinhagen, Germany) was carried out, and composite manufacturing was conducted approximately half an hour after the plasma treatment. A relatively short gap was included to minimize any possible reduction in the effect of the surface treatment. Figure 2 shows the generation of air plasma within the discharge tube and its expulsion through the rotating nozzle onto the sample. The sample was located on the speed controller platform to move under the fixed nozzle. The setup details and operating parameters can be found in a previous study [80]. The fabric-to-torch distance and the platform’s speed were set to 10 mm and 41 mm/s, respectively. Notably, no plasma treatment was necessary for carbon fibers as their adhesion with the PA11 matrix was already excellent, probably due to their original surface treatment.

### 2.3. Composite Manufacturing

**Figure 3:**
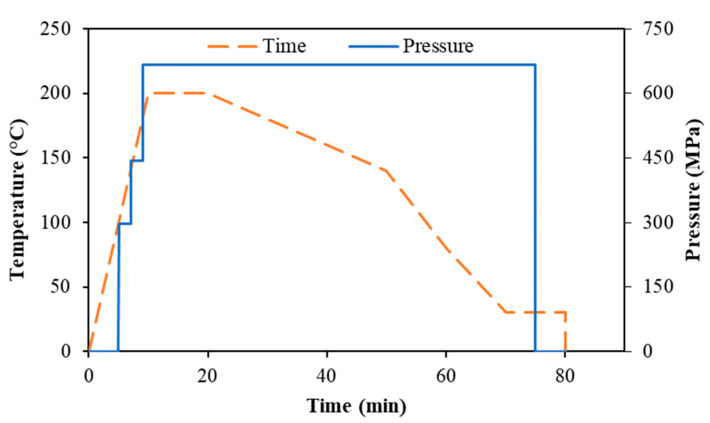
Temperature and pressure profiles for the hot-pressing of the composites.

The laminated composites were produced using a hot-press machine (FontijnePresses TPB374, Barendrecht, The Netherlands) with the cycle presented in Figure 3. The parameters were selected based on various factors, including the thermal and rheological characteristics of the matrix and fibers.

**Figure 4:**
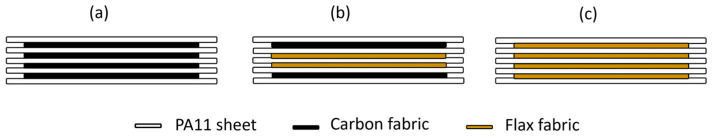
Layup configurations: (**a**) non-hybrid carbon fiber composite ([C_2_]_S_), (**b**) hybrid carbon–flax composite ([CF]_S_), (**c**) non-hybrid flax fiber composite ([F_2_]_S_).

First, two types of non-hybrid composites were manufactured: one with four plies of woven carbon ([C_2_]_S_) and the other with four plies of woven flax ([F_2_]_S_). Then, a hybrid composite was manufactured, composed of two inner plies of woven flax sandwiched between two outer plies of carbon ([CF]_S_). A single PA11 sheet was placed between the fabric layers and on the outside of the layup. Additional details regarding the laminates can be found in Figure 4 and Table 3. The fiber volume fraction of the manufactured composites was determined using a weight-based method.

### 2.4. Peel Test

A floating-roller peel test was performed to determine the force required to separate the laminate layers and evaluate the interlaminar bonding strength. In this test, which is frequently employed to assess the adhesive strength of flexible-to-rigid laminates, one end of the sample is bonded to a rigid substrate, while the other end is left free (flexible end). A controlled force is then applied to the flexible end to peel it away from the substrate or other layers. This entire process is regulated by three rollers that move at the same rate as the material being peeled [82,83]. This method is particularly applicable in cases where traditional peel methods may be less effective. A 3 mm aluminum alloy (EN AW6063-T6) plate was used as a support. The aluminum sheet provided mechanical stability and support during the peel test, preventing specimen bending or deformation. The peel test specimens were prepared following the ASTM-D3167 standard using a thin polytetrafluoroethylene (PTFE) film, approximately 130 mm in length and 20 µm in thickness, at the middle layer of the laminates. This PTFE film was incorporated in the composite layup to create a pre-crack, facilitating the peeling phenomenon. Test specimens with a 25 mm width and a 260 mm length were cut using a band saw and subsequently cleaned with ethanol. A double-sided tape was then applied to the specimen surface for bonding with the aluminum sheet. 

**Figure 5:**
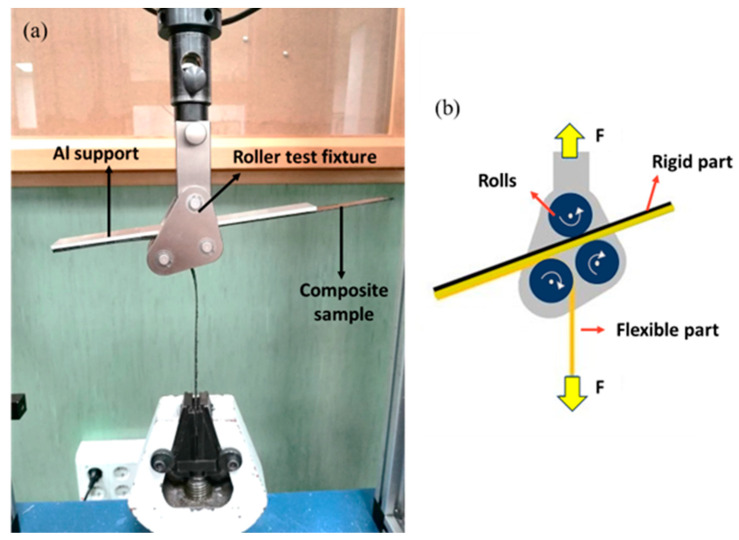
(**a**) The actual setup of the floating roller peel test, showing the physical arrangement and components used; (**b**) schematic diagram illustrating the specimen between the rolls of the floating roller peel test.

**Figure 6:**
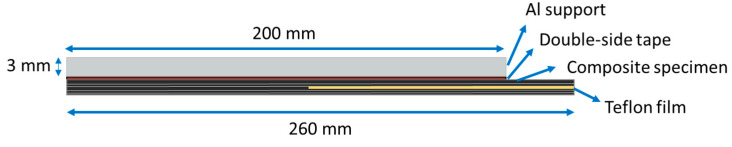
Schematic of the peel test specimen.

Testing was performed utilizing an electromechanical Ibertest machine (Series ELIB20) with a maximum capacity of 20 kN, equipped with a 2 kN load cell at room temperature. The testing speed was set to 5 mm/min. Three specimens were tested for each composite, and the average values are reported with their standard deviation. The applied force and the displacement were measured during the test. The force was averaged over a total of 80 mm displacement starting from 5 mm after the first displacement peak. As depicted in Figure 5 and Figure 6, the PTFE film divided the composites into rigid and flexible parts. The rigid part, bonded to the aluminum plate, was kept between the rollers, while the flexible part was clamped within the lower grip of the testing machine. After the test, the peeled surfaces of the specimens were studied using a Philips XL-33 Scanning Electron Microscope (SEM, Eindhoven, The Netherlands). In preparation for this analysis, a layer of gold was applied to the specimens using a Polaron high-resolution sputter coater. The purpose of this gold coating was to enhance electron conduction and ensure adequate contrast in the SEM micrographs.

### 2.5. Flexural Properties

The flexural properties of the laminates were evaluated in the three-point bending mode following ASTM-D7264 and using a universal testing machine (Instron, 5567, Norwood, MA, USA). The crosshead speed was set to 2 mm/min, with a span-to-thickness ratio of 32:1 and a 1 kN load cell. Specimens were prepared by cutting them to the dimensions of 100 mm × 13 mm using water jet cutting. Five specimens of each composite were tested, and the average values were taken. The flexural strength (σ) and modulus (E) of the composites were determined using the following equations [84]:

(1) $$\sigma =\frac{3F_{\max}L}{2bh^{2}}$$

(2) $$E=\frac{mL^{3}}{4bh^{3}}$$

where L, b, h, F, and m represent the support span length, specimen width, specimen thickness, flexural force, and initial slope of the force–displacement curve, respectively. 

### 2.6. Macro-Hardness Test

The hardness property of the laminates was studied by a macro-Vickers hardness tester (MCI, Toledo, Spain). A square-based pyramid-shaped diamond indenter was applied to the surface of the laminates under controlled ambient conditions with a load of 31.25 kg. The indentation was performed at five different points on each specimen, and the average was noted. The Vickers hardness (Hv) was then determined using the following formula in accordance with ASTM-E384:

(3) $$\mathrm{Hv}=1.854\;\frac{F}{d^{2}}$$

where F and d represent the applied load (kg) and average diagonal of the square impression (mm), respectively. The diagonals after the indentation process were measured by a PCE-MM200 digital optical microscope (PCE Instruments, Albacete, Spain).

## 3. Results

### 3.1. Peel Test

**Figure 7:**
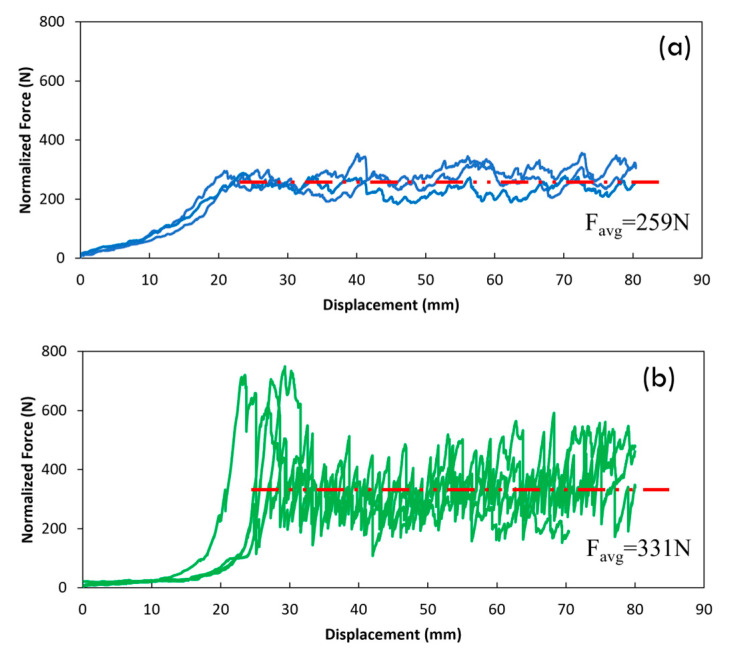
Force–displacement graphs of the peel test for the (**a**) [C_2_]_S_ composites and (**b**) hybrid [CF]_S_ composites, normalized for the laminate fiber volume fraction (the red line shows the average peeling force).

Table 4 and Figure 7 show some results of the peel test. The peel test directly revealed the interlaminar bonding strength between the plies in the laminates. To facilitate a meaningful comparison between the laminates, the values of peel force and strength were normalized based on the fiber volume fractions of the specimens. 

Failure mechanisms during the peel test can include (a) adhesive failure, (b) cohesive failure within the adhesive, and (c) the intralaminar failure of the composite (ILFC) [85,86]. Adhesive failure often occurs at the interface of the matrix and fibers at the interlaminar interface, indicating poor adhesion between the matrix and the fibers. Cohesive failure within the adhesive occurs when the adhesive material itself cannot withstand the applied forces and breaks down internally, leading to adhesive layer rupture. This type of failure is a common mode of failure in situations where a distinct adhesive layer exists between substrates. Intralaminar failure indicates good fiber–matrix adhesion because the failure is cohesive through the plies and does not occur at the fiber–matrix interface [87].

The hybrid composite significantly improved the normalized peel strength (27%) and normalized maximum peel force (115%) compared to the pure carbon fiber composite. Thus, adding flax fibers to the carbon fiber weaves in the middle of the laminate enhanced the interlaminar bonding strength, which can be considered a positive hybridization effect. The force–displacement curve of the hybrid composite fluctuated more than the carbon composite after the maximum force peak. This could be attributed to the interface effect in the hybrid composite. The interfaces between different fiber types and the matrix could influence the overall mechanical behavior. The varying and ununiform interfacial adhesion between the flax, carbon, and matrix led to variations in the bonding strength. These interfacial effects could contribute to localized variations in the force–displacement behavior during the peel test, causing fluctuations in the curve.

**Figure 8:**
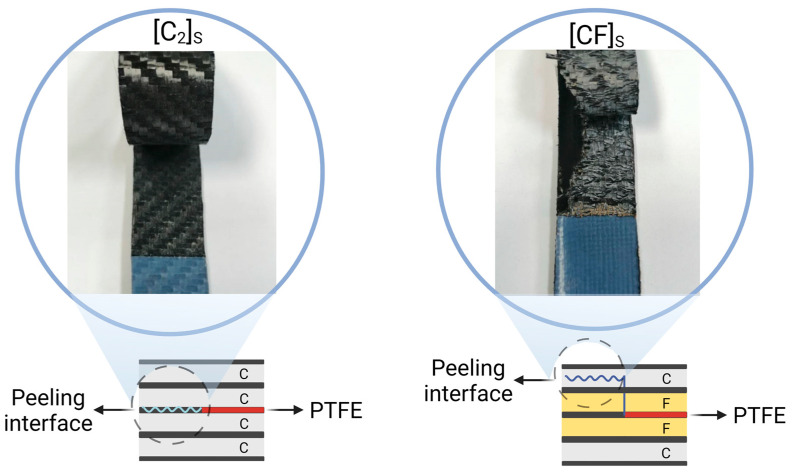
Peeled surfaces of [C_2_]_S_ and hybrid [CF]_S_ composites, indicating the specific carbon ply at which peeling occurred.

The [C_2_]_S_ composite exhibited the adhesive failure mode during peeling at the interface of the carbon ply and matrix. In contrast, the [CF]_S_ composite peeled off through the carbon fiber plies in the flexible part of the specimens with the ILFC failure mode (see Figure 8). The transition from the adhesive mode to the ILFC mode represented a positive hybrid effect. The intralaminar failure of the hybrid composite signified robust fiber–matrix adhesion, as the failure was cohesive across the carbon plies and did not occur at the fiber–matrix interface.

According to Figure 8, the peeling surface in the hybrid composite did not follow the PTFE film; instead, there was a shift of the peeling surface into the first ply of carbon. This could be attributed to the superior impregnation of flax fibers and improved matrix penetration into them. Consequently, the force required to separate the flax–flax and flax–carbon interfaces was higher than the force needed for the intralaminar peeling of the carbon ply. Thus, the peeling surface transitioned from the middle ply to the first ply of the carbon.

**Figure 9:**
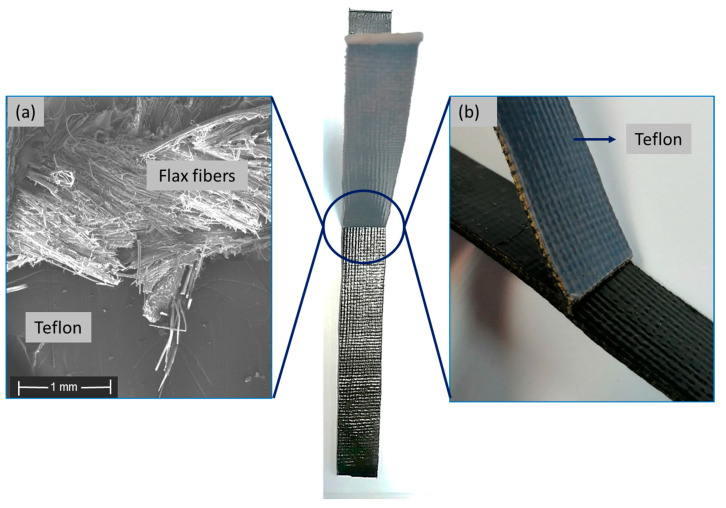
(**a**) SEM image of the failed interface of PTFE and flax fibers, (**b**) image of the failed flax composite specimen during the peel test before any peeling occurred.

The pure flax composite experienced a failure before the initiation of the peeling, occurring specifically in the flexible part at the end of the PTFE film (see Figure 9). Therefore, the peel test could not be completed for this material. The maximum normalized force recorded for the breakage of the [F_2_]_S_ laminate was notably high at 434 N, surpassing the maximum normalized force observed for the [C_2_]_S_ laminate. This higher force implied an improved adhesion of flax fibers with the matrix, demanding more force to initiate the peeling of the plies. Upon further analysis, when this breakage force was normalized by the width of the specimens, it resulted in a value of 17.36 MPa. Remarkably, this value closely aligned with the reported ultimate strength of the PA11 matrix, documented as 20 MPa in the authors’ previous work [79]. The proximity of these values suggests that the failure mechanism could involve the fracture of the matrix. Applying additional force during the peel test could lead to the breakage of the PA11 matrix. The failure of the [F_2_]_S_ laminate, from the fibers’ perspective, could be attributed to the comparatively low flexural strength of the flax composite, as detailed in Section 3.2. Furthermore, flax fibers have a lower tensile stiffness and strength than carbon fibers [88,89]; thus, the pure flax composite was more susceptible to localized stress concentrations, contributing to its premature failure. 

**Figure 10:**
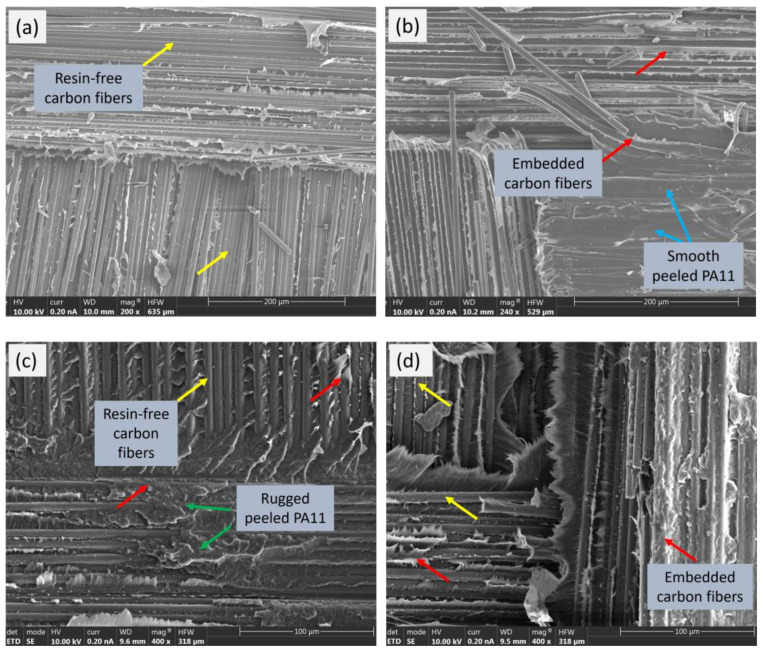
The two peeled surfaces of the (**a**,**b**) [C_2_]_S_ laminate and (**c**,**d**) [CF]_S_ laminate.

Figure 10 shows the peeled surfaces of the [C_2_]_S_ and hybrid [CF]_S_ composites and reveals their internal structures. In Figure 10a,b, which are SEM images from the [C_2_]_S_ specimen, on one peeled surface, resin-free carbon fibers can be observed (yellow arrows), while on the other side, some carbon fibers remain embedded within the PA11 resin (red arrows). This validates the assumption of an adhesive failure mode, as polyamide was observed to be embedded on only one side of the peeled surface. Moreover, the presence of resin-free carbon fibers implies a complete detachment of PA11 from the carbon ply during peeling, suggesting that the adhesion between the carbon fibers and the matrix was relatively weak in those regions. 

As observed in Figure 10c,d, in the case of the hybrid [CF]_S_ composite, both resin-free and embedded carbon fibers were found on both sides of the peeled surfaces.

The presence of both resin-free and embedded carbon fibers suggested that the failure mode was not solely attributed to a complete separation between the fibers and matrix. Instead, it indicated a mixed failure mechanism, where some areas experienced resin detachment while others demonstrated a cohesive failure within the matrix. In addition, the deeper marks on the pulled-out fibers and the greater number of embedded fibers in the matrix after peeling (Figure 10d) in the hybrid composite demonstrated better interlaminar bond strength than in the carbon composite. This could also be confirmed by the flatter and smoother peeled surface of the [C_2_]_S_ composite (see blue arrows) in comparison to the hybrid [CF]_S_ composite, which appeared more rugged (see green arrows). 

In summary, the mechanism of peeling strength improvement in the hybrid composite could be attributed to the positive interaction between the flax and carbon fibers, leading to enhanced interlaminar bonding and a mixed failure mechanism that collectively contributed to superior overall performance in the peel test.

### 3.2. Flexural Properties

**Figure 11:**
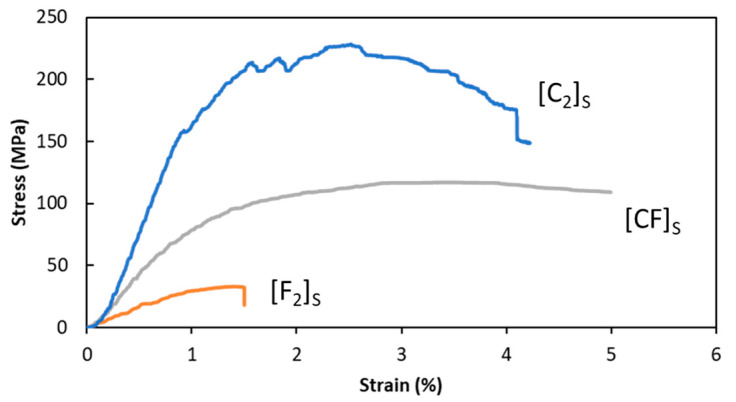
Flexural stress–strain curves of studied composite materials.

The average flexural properties of the composite laminates and their stress–strain curves are reported in Table 5 and Figure 11, respectively. All composites displayed nonlinear behavior with a prolonged stress–strain curve. As expected, the hybridization of carbon and flax fibers led to an improvement in flexural properties when compared to the pure flax fiber composite. This enhancement substantially increased the modulus (212%) and flexural strength (265%). The hybrid composite still fell short of the superior values of the pure carbon composite. A similar trend has been observed in other studies [28,89,90], which have shown that carbon fibers have higher intrinsic mechanical properties compared to flax fibers.

Additionally, during the flexural test, the top surface experienced compressive stresses, while the bottom surface experienced tensile stresses, and the middle part of the specimen experienced shear stresses. Moreover, using multiple layers of fabrics in a hybrid configuration could result in increased shear deformation between the different layers of the laminated composite, leading to stress concentration. This stress concentration increased the possibility of early failure in the layers and consequently contributed to the reduced strength of the hybrid composite. By replacing two layers of carbon with flax in the hybrid composite, the overall carbon fiber volume fraction decreased from 30% to 10%, resulting in a reduced fraction of high-strength carbon fibers. Consequently, this decrease in carbon fiber content led to a lower overall flexural strength and modulus in the hybrid composite. Furthermore, the bond quality between the fabrics and the matrix is crucial in load transfer and overall mechanical performance. Carbon fibers typically exhibit better adhesion with the matrix compared to flax fibers. This difference in interfacial bonding can cause variations in stress transfer and subsequently affect the overall strength of the composite.

**Figure 12:**
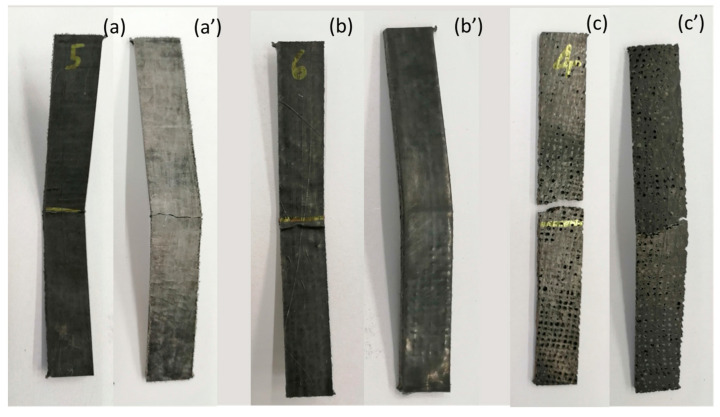
Fractured flexural samples after three-point bending test: (**a**) compression side of [C_2_]_S_ composite; (**a’**) tension side of [C_2_]_S_ composite; (**b**) compression side of [CF]_S_ composite; (**b’**) tension side of [CF]_S_ composite; (**c**) compression side of [F_2_]_S_ composite; (**c’**) tension side of [F_2_]_S_ composite.

The hybrid composite exhibited a notable characteristic in its stress–strain curve, as it demonstrated prolonged behavior without failure until reaching 5% strain. This indicated the composite’s ability to withstand higher strains without experiencing failure (Figure 12b,b’), surpassing both the carbon and flax composites in this regard. In essence, the hybrid composite displayed a 34% increase in strain at maximum strength compared to the pure carbon composite, highlighting its superior ductility. These findings show that [CF]_S_ hybrid laminate composites have promising prospects as lightweight and eco-friendly substitutes for pure carbon composites, especially in scenarios demanding high levels of ductility.

The presence of flax fibers in the hybrid composite could lead to crack deflection. When cracks propagated in the composite, the less stiff and more ductile flax fibers could cause the cracks to change direction or slow down. This could effectively increase the overall failure strain of the laminate as compared to pure carbon, where cracks might propagate more easily. However, in the pure flax laminate, this advantage was not as pronounced due to the absence of outer carbon plies that would provide additional support and stiffness. Moreover, in the pure flax laminate, the flax fabrics, located on the outer layers, were more susceptible to buckling when subjected to compressive forces, which could limit their ability to contribute to crack deflection. Buckling could concentrate stress, potentially leading to fiber breakage or other damage that could ultimately result in fracture, as can be seen in Figure 12c.

### 3.3. Macro-Hardness Test

**Figure 13:**
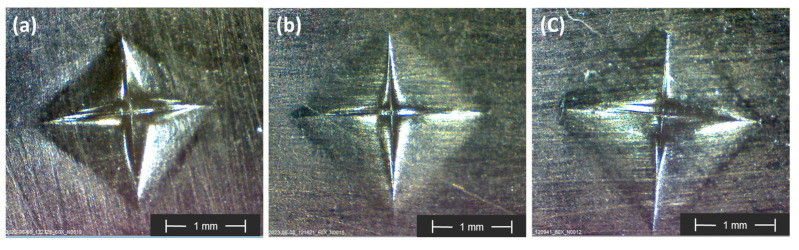
Indentation impressions after Vickers hardness test obtained by digital microscope: (**a**) [C_2_]_S_ composite; (**b**) [CF]_S_ hybrid composite; (**c**) [F_2_]_S_ composite.

The indentation impressions and corresponding hardness values for different laminates are presented in Figure 13 and Table 6, respectively. 

The reference composite [F_2_]_S_ exhibited the lowest macro-hardness of the tested composites because of the lower mechanical strength of flax fibers compared to carbon fibers. On the other hand, the hybrid composite [CF]_S_ demonstrated the highest macro-hardness among all the composites. The results of the [C_2_]_S_ and [CF]_S_ composites were also statistically analyzed by *t*-tests at a 5% significance level. The *t*-tests did not show statistically significant differences in the macro-hardness between the hybrid and [C_2_]_S_ composites (*p*-value > 0.05). This implies that the replacement of two internal carbon plies with flax plies did not adversely affect the laminate’s hardness properties, particularly when the skin layer was consistent between both laminates and the indenter did not penetrate beyond the first ply.

The enhancement in hardness in the [CF]_S_ composite compared to the [F_2_]_S_ composite could be attributed to the inclusion of high-strength carbon fibers on the outer ply, which reduced the penetration of the diamond indenter into the surface. This resulted in a 28% increase in macro-hardness compared to the pure flax composite.

## 4. Conclusions

In conclusion, the woven carbon–flax/PA11 hybrid bio-composites with a sandwich configuration ([CF]_S_) showed remarkable improvements in mechanical properties compared to the pure flax and carbon composites. Notably, they exhibited significant enhancements in peel strength (27%) and maximum peel force (115%) compared to the pure carbon composite. These improvements could be attributed to the positive interaction between flax and carbon fibers, leading to improved interlaminar bonding and a mixed failure mechanism. Furthermore, the hybrid composite displayed remarkable increases in flexural modulus (212%) and flexural strength (265%) when compared to the pure flax composite. While the hybrid composite may not have reached the exceptional values of the pure carbon composite, it did exhibit a notable advantage in terms of ductility. It showed a 34% increase in strain at maximum strength, highlighting enhanced ductile properties attributed to the crack deflection mechanism introduced by the flax fibers. Notably, the reference flax composite exhibited the lowest hardness due to the weaker mechanical strength of flax fibers. Furthermore, the substitution of two carbon plies with flax plies did not significantly compromise the laminate’s macro-hardness properties since both hybrid and carbon laminates exhibited comparable hardness values. 

In summary, this hybrid bio-composite offers a promising compromise between the unique properties of carbon and flax fibers, making it a valuable material for applications requiring a balance of strength, hardness, bonding, and ductility.

## Figures and Tables

**Table 2:** Average properties of the flax and carbon fibers used herein.

Properties	Flax	Carbon
Density (g/cm^3^)	1.5	1.7
Diameter (µm)	20	4
Tensile modulus (GPa)	12	240
Tensile strength (MPa)	106	4100
Failure strain (%)	1.5	1.7

**Table 3:** Properties of the produced laminated composites.

Layup	Average Thickness (mm)	Fiber Volume Fraction (%)		
		Flax (V_f_)	Carbon (V_c_)	V_f_ + V_c_
[C_2_]_S_	1.60 ± 0.06	0	30	30
[CF]_S_	2.06 ± 0.03	30	10	40
[F_2_]_S_	2.32 ± 0.04	42	0	42

**Table 4:** Results of the peel test along with associated failure modes.

Layup	Normalized Maximum Force (N)	Normalized Peeling Strength (N/mm)	Failure Mode
[C_2_]_S_	323 ± 28	10.4 ± 0.9	Adhesive
[CF]_S_	697 ± 52	13.2 ± 0.4	ILFC
[F_2_]_S_	434 ± 4	_	Matrix/fiber breakage

**Table 5:** Flexural test results of the studied composite materials.

Layup	E (GPa)	Max Strength (Mpa)	Strain at Max Stress (%)
[C_2_]_S_	19 ± 2	217 ± 7	2.6 ± 0.2
[CF]_S_	10 ± 1	117 ± 5	3.5 ± 0.2
[F_2_]_S_	3.2 ± 0.3	32 ± 2	1.4 ± 0.4

**Table 6:** Vickers hardness of studied composites.

Layup	Vickers Hardness (Hv)
[C_2_]_S_	16.99 ± 0.76
[CF]_S_	17.84 ± 0.74
[F_2_]_S_	13.90 ± 1.12

## Data Availability

The datasets generated and/or analyzed during the current study are available from the corresponding author upon reasonable request.
